# Rapid diagnostic tests for malaria and health workers’ adherence to test results at health facilities in Zambia

**DOI:** 10.1186/1475-2875-13-166

**Published:** 2014-05-02

**Authors:** Christine Manyando, Eric M Njunju, Justin Chileshe, Seter Siziya, Clive Shiff

**Affiliations:** 1 Tropical Diseases Research Centre, Ndola, Zambia; 2 Copperbelt University, School of Medicine, Ndola, Zambia; 3 Department of Molecular Microbiology and Immunology, Johns Hopkins Bloomberg School of Public Health, Baltimore, MD, USA

**Keywords:** Rapid diagnostic tests (RDTs), Malaria, Febrile illness, Microscopy, Anti-malarials, Health centre, Elimination

## Abstract

**Background:**

In Zambia, there has been a large scaling up of interventions to control malaria in recent years including the deployment of rapid diagnostic tests (RDTs) to improve malaria surveillance data as well as guide malaria treatment in health facilities. The practical challenge is the impact of RDT results on subsequent management of patients. This study explored the role of RDTs in malaria diagnosis and the health workers’ adherence to test results.

**Methods:**

An observational prospective study was carried out at health centres in four districts, namely Chibombo, Chingola, Chipata, and Choma. Children under the age of five years with history of fever were recruited and the clinicians’ use of RDT results was observed to establish whether prescriptions were issued prior to the availability of parasitological results or after, and whether RDT results influenced their prescriptions.

**Results:**

Of the 2, 393 recruited children, 2, 264 had both RDT and microscopic results. Two in three (68.6%) children were treated with anti-malarials despite negative RDT results and almost half (46.2%) of these were prescribed Coartem^®^. Only 465 (19.4%) of the 2,393 children were prescribed drugs before receiving laboratory results. A total of 76.5% children were prescribed drugs after laboratory results. Children with RDT positive results were 2.66 (95% CI (2.00, 3.55)) times more likely to be prescribed anti-malarial drugs. Children who presented with fever at admission (although history of fever or presence of fever at admission was an entry criterion) were 42% less likely to be prescribed an anti-malarial drug compared to children who had no fever (AOR = 0.58; 95% CI (0.52, 0.65)). It was noted that proportions of children who were RDT- and microscopy-positive significantly declined over the years from 2005 to 2008.

**Conclusions:**

RDTs may contribute to treatment of febrile illness by confirming malaria cases from non-malaria cases in children under the age of five. However, the adherence of the health workers to prescribing anti-malarials to only RDT-positive cases at health facility level will still require to be explored further as their role is crucial in more precise reporting of malaria cases in this era towards malaria elimination as the target.

## Background

The current global policy recommendation is universal access to parasite-based diagnosis of malaria-like febrile illness [1] due to the use of artemisinin-based combination therapy (ACT), as the first-line anti-malaria treatment for *Plasmodium falciparum* infection in most endemic countries. ACT is expensive and relies on an insecure supply of raw materials. It is also documented that non-malarial febrile illnesses exert higher childhood mortality across malaria-endemic countries than malaria [2]. It is for this reason that rapid diagnostic tests (RDTs) have an important role to play in targeting treatment with ACT to malaria cases, and non-malarial cases to other forms of management.

Malaria continues to be a disease of public health importance in Zambia. There has been a large scaling up of interventions to control malaria in recent years [3]. In 2009, 2.9 million cases and about 3,862 malaria attributable deaths were reported in a population of 12 million, down from 3.3 million reported cases and 9,369 deaths [4]. This is because, in 2004, Zambia began to progressively introduce ACT in response to growing and widespread resistance to sulphadoxine-pyrimethamine (SP) and chloroquine (CQ) with full national scale reached by early 2005 [5]. At the time of the shift to ACT much diagnosis was based on non-specific symptoms rather than confirmed malaria parasitaemia; consequently, actual malaria incidence at health facilities remained unquantified and many anti-malarial treatments were misdirected [5].

Zambia was faced with poor malaria surveillance data caused by inadequate laboratory diagnostic facilities in most parts of the country at the time of introduction of ACT. This, coupled with substantial higher cost of Coartem^®^ compared with SP and CQ, compelled the National Malaria Control Programme (NMCP) to introduce RDTs, firstly as pilot in some districts in 2005 and 2006. Scale up at national level was achieved in 2009, when Zambia made the transition to a strategy of full reporting and treatment using only parasitologically confirmed diagnosis nationwide [5]. Laboratory diagnosis is now mandatory, where capacity exists, before anti-malarial treatment, although in some cases stock outs of reagents or test kits may prevent full enactment of this policy [6].

The practical challenge is that the impact of RDT results on subsequent management of patients varies. The impact of RDTs on anti-malaria consumption has been demonstrated widely [5,7,8] but a number of field trials have also demonstrated poor adherence to results, therefore reducing the potential of RDTs to improve disease management [9-12]. RDT uptake and utilization remains low in public health settings; evidence shows that providing RDTs in the context of formal health care settings may have limited impact on clinicians’ prescribing behaviour [13,14], yet the cost benefits of improved diagnosis can only be realized when treatment is consistent with test results [15]. Most of the data available on the usefulness of RDTs in many settings has been generated in research settings with limited consideration of their impact on anti-malarial drug consumption in real-life settings [16]. It is hoped that RDTs may contribute to changing malaria reporting practices and the usage of ACT in Zambia and that over time RDTs can change clinical practice and move the case definition of malaria (meaning all suspected malaria cases or all fever cases) in routine health systems to a definition of malaria that is routinely based on laboratory-confirmed parasitaemia combined with clinical symptoms [5].

A study was conducted to explore the role of RDTs in order to determine the adherence (i.e. the compliance to treat every patient with positive RDT result with an anti-malarial and not to treat when the RDT is negative) by health workers to test results for the management of febrile illness in children under five years of age in four districts of different epidemiological zones. The study was designed to compare the extent of excessive use of Coartem^®^ between the clinical paradigm and the RDT with the standard thick blood film microscopy.

## Methods

### Study area

The study was conducted from 2005 to 2008 in four districts, namely Chingola, Chibombo, Choma, and Chipata which cover a range of transmission intensity. In each one of these districts, two health facilities were selected. In Chingola, the study was conducted at Chawama clinic and Clinic 1; in Chibombo, at Liteta Hospital and Chisamba clinic. In Choma, the study was conducted at Shampande clinic and Choma General Hospital and in Chipata at Kapata Clinic and Chipata Health Affiliated Clinic (HAC).

At the time of the conduct of the study, Chingola, in the Copperbelt province of Zambia with a population of 177,445 (2000 Census), was a district in an area of high malaria transmission with prevalence in the range of 35% in the dry season between May and October and 70% in the wet season, November to April, in children under the age of five years attending outpatient care.

Choma with a population of 203,305 (2000 Census) in the Southern Province of Zambia, on the other hand, was in an area of unstable but severe and irregular transmission. The prevalence of malaria in children under five years of age ranged between 33 and 68%. Chipata situated in the Eastern Province of Zambia with a population of 362,132 (2000 Census) was in an area of stable transmission with prevalence rates ranging from 35 to 70%. Chibombo with a population of 242,380 (2000 Census) in the central part of the country was in a medium transmission zone with prevalence ranging from 5 to 45%.

### Study population

The study population was children under the age of five years, weighing more than 10 kg and residing within the catchment area of the recruiting health facility. These children were included in the study based on history (or presence) of fever and being clinically diagnosed as having malaria by the attending clinician at the health facility.

### Study procedure

The study clinician examined each child who was eligible to enter the study, took history and explained the study protocol to the parent or guardian of the child. Written consent was obtained from all parents or guardians who agreed to have their children participate in the study. Two scenarios were observed and noted by study investigators. The clinician, at his/her own discretion, prescribed an anti-malarial before the patient went to the laboratory for an RDT or at the clinician’s discretion, the patient was sent to the laboratory for a malaria test and anti-malarial prescription was given only after RDT results were made available. The study investigators (blinded to the clinician) took note of the adherence of the attending clinician to await a RDT result before prescribing an anti-malarial or prescribing of the anti-malarial before the availability of the RDT results.

All patients were advised to return for review on day 3 and 7. On these days, only blood for microscopy was collected to ensure the patients were fully treated and recovered before being discharged from the sick list.

In the laboratory, a finger prick was made on the patient to collect blood for RDT (ICT Malaria Combo Cassette Test Ref: ML02 25 Test Kit from ICT Diagnostics, Bedfordview, 2008, South Africa) and microscopy. Only the RDT result was available to the clinician (for those patients whom prescription awaited laboratory results). Blood slides were stained and read later within the health facilities and results entered in the log books.

### Training

Consistency of operation was required for the study to be conducted in a uniform manner across the country; therefore an element of training study staff was crucial before the study was initiated. This was carried out at a neutral venue, under the coordination of the investigators. The training took seven days and the following comprised the content of the training: to administer the study information and informed consent form; to fill out details on a structured case record form (CRF); how to follow step-by-step the patient flow chart and the aspect of counselling the patient about post-treatment follow-up. The latter included what the parent/guardian should do in the event that the child’s condition post-consultation with study staff worsened; information regarding what treatment the child had been prescribed and counselling for the third and seventh day follow-up visits.

### Data management and analysis

The data were entered and initially analysed in EPI INFO (version 3.3.2 of 2005). Consistency and range checks were used to edit the data. Further analysis was conducted using SPSS (version 17.0 of 2008). The Cohen’s Kappa statistic with its 95% confidence interval was used to measure the extent of agreement between RDT and microscopic results. Multivariate logistic regression analysis was conducted to determine independent factors associated with the outcome. Adjusted odds ratios (AORs) and their 95% CIs were reported.

### Variables

The variables used for analysis included the following: fever (axillary temperature of 37.4 degrees Celsius or higher), history of fever, laboratory result by RDT, anti-malarial drugs prescribed (Coartem^®^, SP or quinine), prescription given (before or after).

### Ethical considerations

The study was conducted after approval from the Tropical Diseases Research Centre (TDRC) Ethics Review Committee. Technical support throughout the proposal development and planning phase came from MIM/WHO/TDR.

## Results

A total of 2,393 children participated in the study of which 52.1% were males. The median age was 25 (Q_1_ = 17, Q_3_ = 36) months. Altogether 2,264 children had both the RDT and microscopic results: 640 were from Chibombo, 584 from Chingola, 398 from Chipata and 642 from Choma.

Overall, 68.6% of the children were treated for malaria despite having negative RDT results. There were a total 44.9% of the children from Chibombo, 69.5% from Chingola, 97.0% from Chipata, and 57.2% from Choma (Table 1). Almost half (46.2%) of the children across all districts who were negative on RDT results were given Coartem^®^.

**Table 1:** Treatment given regardless of rapid diagnostic test results

	**RDT results**		
**District/**	**Positive**	**Negative**	**Total**
**Treatment given**	**n (%)**	**n (%)**	**n (%)**
**Chibombo**	**Total = 47 (16.1)**	**Total = 245 (83.9)**	**Total = 292 (100)**
Coartem^®^	36 (76.6)	85 (34.7)	121 (41.4)
SP	5 (10.6)	25 (10.2)	30 (10.3)
Quinine	1 (2.1)	2 (0.8)	3 (0.01)
None	5 (10.6)	133 (54.3)	138 (47.3)
**Chingola**	**Total = 71 (13.1)**	**Total = 470 (86.9)**	**Total = 541 (100)**
Coartem^®^	54 (76.1)	214 (45.5)	268 (49.5)
SP	7 (9.9)	104 (22.1)	111 (20.5)
Quinine	5 (7.0)	9 (1.9)	14 (2.6)
None	5 (7.0)	143 (30.4)	148 (27.4)
**Chipata**	**Total = 39 (11.6)**	**Total = 296 (88.4)**	**Total = 335 (100)**
Coartem^®^	22 (56.4)	205 (69.3)	227 (67.8)
SP	5 (12.8)	73 (24.7)	78 (23.3)
Quinine	10 (25.6)	9 (3.0)	19 (5.7)
None	2 (5.1)	9 (3.0)	11 (3.3)
**Choma**	**41 (12.6)**	**283 (87.3)**	**324 (100)**
Coartem^®^	32 (78.0)	94 (33.2)	126 (38.9)
SP	6 (14.6)	68 (24.0)	74 (22.8)
Quinine	3 (7.3)	0 (0)	3 (1.0)
None	0 (0)	121(42.8)	121 (37.3)
**All districts**	**Total = 198 (13.3)**	**Total = 1,294 (86.7)**	**Total = 1,492 (100)**
Coartem^®^	144 (72.7)	598 (46.2)	742 (49.7)
SP	23 (11.6)	270 (20.9)	293 (19.6)
Quinine	19 (9.6)	20 (1.5)	39 (2.6)
None	12 (6.1)	406 (31.4)	418 (28.0)

As investigators observed the prescribers (in a blinded manner) and noted those who prescribed anti-malarials prior to the availability of laboratory results, it was observed that overall, 465 (19.4%) out of 2,393 children were prescribed anti-malarial drugs *before* receiving laboratory results. Of the children who received anti-malarial prescription *before* receiving laboratory results, 84.0% were later found to be RDT result negative; 77.8% had no fever; 54.0% received Coartem^®^ while 36.1% received SP, 5.4% quinine and 4.5% did not receive any anti-malarial drug (Table 2).

**Table 2:** Distributions of fever, anti-malarial drugs given and rapid diagnostic test results by anti-malarial drug prescription after laboratory results

**Factor**	**Anti-malarial drug prescribed after laboratory result**	
	**Yes**	**No**
	**n (%)**	**n (%)**
***Fever***		
Yes	888(59.3)	103 (22.2)
No	610 (40.7)	360 (77.8)
**Total**	**1,498 (100)**	**463 (100)**
***Anti-malarial drug given***		
Coartem^®^	743 (49.6)	251 (54.0)
SP	296 (19.7)	168 (36.1)
Quinine	41 (2.7)	25 (5.4)
None	419 (28.0)	21 (4.5)
**Total**	**1,499 (100)**	**465 (100)**
***RDT result***		
Positive	200 (13.4)	74 (16.0)
Negative	1,295 (86.6)	389 (84.0)
**Total**	**1,495 (100)**	**463 (100)**

Table 3 shows variations in the proportions of children who were prescribed anti-malarials *before* receiving laboratory results by district. About half (53.6%) of the children from Chibombo received anti-malarial prescriptions before receiving laboratory results, while in Chipata 0.9% of the children were prescribed anti-malarials before receiving laboratory results.

**Table 3:** Proportions of children prescribed anti-malarials against rapid diagnostic test results per district

**District**	**Total (%)**	**Prescribed anti-malarial drugs before laboratory results**	
		**Yes**	**No**
		**n (%)**	**n (%)**
Chibombo	647 (100)	347 (53.6)	300 (46.4)
Chingola	606 (100)	32 (5.3)	574 (94.7)
Chipata	461 (100)	4 (0.9)	457 (99.1)
Choma	679 (100)	83 (12.2)	596 (87.8)
Total		466	1,927

A total of 1,498 (76.5%) of the 1,958 children were given anti-malarial prescriptions after receiving laboratory results (Table 4). No information was recorded on six children. While children who had only fever or had fever with RDT negative result were less likely to receive a prescription, children who had RDT positive result, had fever and had RDT positive result, had no fever and had RDT positive result, were more likely to receive anti-malarial prescription.

**Table 4:** Distributions of fever, anti-malarial drugs given and rapid diagnostic test results by anti-malarial drug prescription after laboratory results

**District**	**Total (%)**	**Fever**	**Prescribed anti-malarial drugs after laboratory results**	
			**Yes**	**No**
			**n (%)**	**n (%)**
Chibombo	637 (100)	Yes	266 (90.8)	83 (24.1)
		No	27 (9.2)	261 (75.9)
		**Total**	**293 (100)**	**344 (100)**
Chingola	571 (100)	Yes	394 (73.1)	15 (46.9)
		No	145 (26.9)	17 (53.1)
		**Total**	**539 (100)**	**32 (100)**
Chipata	345 (100)	Yes	140 (41.1)	2 (50.0)
		No	201 (58.9)	2 (50.0)
		**Total**	**341 (100)**	**4 (100)**
Choma	408 (100)	Yes	88 (27.1)	3 (3.6)
		No	237 (72.9)	80 (96.4)
		**Total**	**325 (100)**	**83 (100)**

On multivariate analysis, RDT results and fever were significantly associated with prescribing anti-malarial drugs after knowing laboratory results (Table 5). Children who had RDT result positive were 2.66 (95% CI (2.00, 3.55)) times more likely to be prescribed anti-malarial drugs than children who were RDT result negative. Meanwhile, children who presented with fever at admission were 42% (AOR = 0.58; 95% CI (0.52, 0.65)) less likely to be prescribed anti-malarial drugs compared to children who had no fever (although of course had history of fever at presentation).

**Table 5:** Factors independently associated with receiving prescription

**Factor**	**AOR (95% CI)**
RDT result	
Positive	2.66 (2.00, 3.55)
Negative	1
Fever	
Present	0.58 (0.52, 0.65)
Absent	1

**Figure 1:**
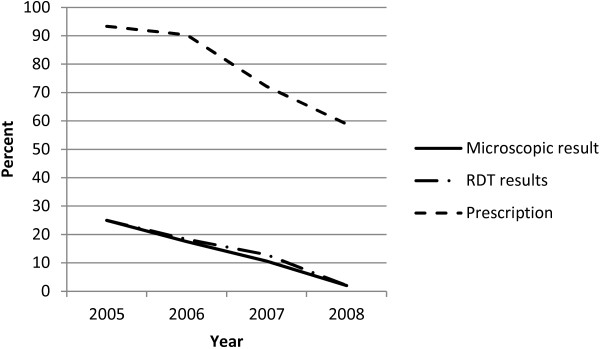
Microscopic and rapid diagnostic test results and prescription over time.

Changes in microscopic and RDT results as well as proportions being prescribed anti-malarials for positive laboratory results over time are shown in Figure 1. In all the three parameters, proportions of children who were positive and prescribed anti-malarials significantly declined over time (microscopic results: slope = -7.590, 95% CI: -8.615, -6.565; RDT results: slope = -7.440, 95% CI: -10.968, -3.912; prescribed anti-malarials: slope = -12.130, 95% CI: -21.391, -2.869).

## Discussion

The World Health Organization (WHO) recommends confirmatory diagnosis before treatment in all settings where a diagnostic test is or can be made available. The Ministry of Health in Zambia revised the national guidelines in 2009–2010 to mandate parasitological confirmation of malaria based on microscopy or RDTs in the public and private sectors, with RDTs to be used where microscopy was not available, or where RDT use on an outpatient basis by non-laboratory staff would reduce laboratory workloads [17].

This study was carried out before the guidelines were enforced and the assessment of the prescribing practices between availability of laboratory results and anti-malarial prescriptions indicated that only 19.4% of children were prescribed medication prior to receiving laboratory results. This category of clinicians did not wait for the availability of results before prescribing anti-malarials. However, for this smaller number, over 50% of the prescriptions given (i.e. 9.7% of the children) were Coartem^®^ and lesser of other anti-malarials. Over 77.8% of these patients were not febrile. On counterchecking the parasitological results of the patients who received anti-malarials prior to laboratory results, 84.0% were RDT-negative who should not otherwise have received anti-malarial medication. One assumption that could be made is that this category of health workers (who prescribed for the 19.4% patients) may perhaps not trust the laboratory results yet, or that they remain with the orientation of prescribing an anti-malarial under the Integrated Management of Childhood Illness (IMCI) guidelines (although the absence of fever in most cases contradicts this). Chibombo district was most affected by prescriber preference to treat with an anti-malarial before the laboratory results (53.6%). It’s encouraging however, to generally note that the number of children in this study who were treated before laboratory results were fewer.

Of the children whose laboratory results were available before prescriptions were given, those whose results were RDT-positive were 2.66 times more likely to receive an anti-malarial. Nonetheless, for the children who had fever (who presented at the health facility with raised temperature), they were 42% less likely to be prescribed anti-malarial drugs compared to their counterparts who had no fever. This should be interpreted with caution as all children recruited in this study had either a history of fever or were found to be febrile at the time of inclusion in the study. As regards the rationalization of treatment on the basis of fever, this study has confirmed that febrile illness was not the basis for prescription of an anti-malarial *per se*. Febrile, parasite-negative patients were still prescribed an anti-malarial as much as afebrile patients with RDT-negative results.

Laboratory confirmation of parasite infection is important to the management of malaria and other febrile illness because to properly manage febrile illness, it is necessary for clinicians to accurately know the actual infection status of each patient. As RDTs may be the only available tool for very remote areas in Zambia, the issue of clinicians trusting the results is paramount if confirmation of parasite infection has to remain the basis for prescribing an anti-malarial.

As a follow-up to the observations by Yukich *et al.*, that provider non-compliance with test results can lead to reductions in the ability of RDT introduction to reduce ACT consumption and concluded that this may not be a major problem in Zambia [5], findings from the eight health centres in the four districts has found a situation to the contrary. In terms of timing, the two studies were conducted at the same time. Overall, over 70% of children were treated for malaria after the availability of RDT results. The treatments were ACT and other anti-malarials but ACT was more prescribed compared to other anti-malarials whether RDT results were positive or not.

The high levels of malaria clinical diagnoses with anti-malarial prescription in this study were observed across all the districts despite the varied malaria prevalence. In areas of high malaria transmission, where clinical diagnosis may be a marginally more effective predictor of malaria infection, this practice would hardly raise concern. The findings of this study do not allow for the fact that the roll-out of RDTs may contribute to the changing of malaria case definition to mean parasitological malaria and not suspected malaria cases or fever cases.

The management of fever in children less than five years in the districts where this study was conducted may be interpreted to be non-compliant to RDT results as there were less actual parasite-positive malaria patients than were treated. The report of Njama-Meya *et al.* is reassuring in that the incidence of malaria in febrile patients left untreated after seven days post a negative result was significantly lower than the incidence of malaria in the whole cohort [18]. This finding supports the policy of restricting anti-malarials to parasitologically confirmed cases (as was also the findings of Msellem *et al*., and others [19-25]).

The over-prescription pattern found in Chibombo and the RDT compared to microscopy discrepancies reported in Chipata (based on microscopy and RDT performance as validated by other studies [9,26,27]) may indicate a need for further orientation of the clinicians and laboratory technicians in Chibombo and Chipata, respectively.

It is important to underscore that over time in this study, there was a decline in the number of positive malaria cases and anti-malarial prescriptions, confirming the already noted reduction in malaria prevalence countrywide. Therefore, emphasizing the importance for RDT result adherence to allow for interpretation of malaria prevalence over time and treatment of actual malaria cases. This would avoid presumptuous prescriptions and thereby missing febrile cases that would require further investigations and appropriate treatment.

## Conclusion

RDTs may contribute to treatment of febrile illness in children under the age of five years for malaria and non-malaria cases. However, compliance by the health workers to test results at health facility level will still require further exploration, as their role is crucial in more precise reporting of malaria cases in this era towards the target of malaria elimination. The laboratory staff at health facility level may still require in-service re-orientation in microscopy where these facilities are available for correct interpretation on trends in malaria epidemiology in Zambia.

## Competing interests

The authors declare that they have no competing interests.

## Authors’ contributions

All authors read and approved the final manuscript. CM, EMN and CS participated in the conception and design of the study and protocol development. CM, JC and EMN participated in the training. CM, EMN and JC coordinated the data collection, management and participated in the manuscript writing. JC carried out the analyses which were validated by SS who provided overall direction on the analysis. CS conceived the study and provided technical oversight to the design of the study.
